# Neuroprotective effect of a new DJ-1-binding compound against neurodegeneration in Parkinson's disease and stroke model rats

**DOI:** 10.1186/1750-1326-6-48

**Published:** 2011-07-08

**Authors:** Yoshihisa Kitamura, Shotaro Watanabe, Masanobu Taguchi, Kentaro Takagi, Takuya Kawata, Kazuko Takahashi-Niki, Hiroyuki Yasui, Hiroshi Maita, Sanae MM Iguchi-Ariga, Hiroyoshi Ariga

**Affiliations:** 1 Department of Neurobiology, Kyoto Pharmaceutical University, Kyoto 607-8414, Japan; 2 Graduate School of Agriculture, Hokkaido University, Sapporo, Japan; 3 Department of Analytical and Bioinorganic Chemistry, Kyoto Pharmaceutical University, Kyoto 607-8414, Japan; 4 Graduate School of Pharmaceutical Sciences, Hokkaido University, Sapporo, Japan

## Abstract

**Background:**

Parkinson's disease (PD) and cerebral ischemia are chronic and acute neurodegenerative diseases, respectively, and onsets of these diseases are thought to be induced at least by oxidative stress. PD is caused by decreased dopamine levels in the substantia nigra and striatum, and cerebral ischemia occurs as a result of local reduction or arrest of blood supply. Although a precursor of dopamine and inhibitors of dopamine degradation have been used for PD therapy and an anti-oxidant have been used for cerebral ischemia therapy, cell death progresses during treatment. Reagents that prevent oxidative stress-induced cell death are therefore necessary for fundamental therapies for PD and cerebral ischemia. DJ-1, a causative gene product of a familial form of PD, PARK7, plays roles in transcriptional regulation and anti-oxidative stress, and loss of its function is thought to result in the onset of PD. Superfluous oxidation of cysteine at amino acid 106 (C106) of DJ-1 renders DJ-1 inactive, and such oxidized DJ-1 has been observed in patients with the sporadic form of PD.

**Results:**

In this study, a compound, comp-23, that binds to DJ-1 was isolated by virtual screening. Comp-23 prevented oxidative stress-induced death of SH-SY5Y cells and primary neuronal cells of the ventral mesencephalon but not that of DJ-1-knockdown SH-SY5Y cells, indicating that the effect of the compound is specific to DJ-1. Comp-23 inhibited the production of reactive oxygen species (ROS) induced by oxidative stress and prevented excess oxidation of DJ-1. Furthermore, comp-23 prevented dopaminergic cell death in the substantia nigra and restored movement abnormality in 6-hydroxyldopamine-injected and rotenone-treated PD model rats and mice. Comp-23 also reduced infarct size of cerebral ischemia in rats that had been induced by middle cerebral artery occlusion. Protective activity of comp-23 seemed to be stronger than that of previously identified compound B.

**Conclusions:**

The results indicate that comp-23 exerts a neuroprotective effect by reducing ROS-mediated neuronal injury, suggesting that comp-23 becomes a lead compound for PD and ischemic neurodegeneration therapies.

## Background

Parkinson's disease (PD) is a chronic neurodegenerative disease caused by dopaminergic cell death, and genetic and environmental factors are thought to affect the onset of PD. Cerebral infarction and stroke are acute neurodegenerative diseases caused by ischemic injury. Onsets of these diseases are thought be induced at least by oxidative stress, but the precise mechanisms are still not known. Although a precursor of dopamine, inhibitors of dopamine degradation and dopamine releasers have been used for PD therapy and an anti-oxidant have been used for cerebral infarction and stroke, cell death progresses during treatment. Identification of compounds or proteins that inhibit oxidative stress-induced neuronal cell death is necessary.

DJ-1 was first identified by our group as a novel oncogene product [1] and later found to be a causative gene product of a familial form of PD, PARK7 [2]. DJ-1 plays roles in transcriptional regulation [3-9] and anti-oxidative stress reaction [10-13], and loss of its function is thought to result in the onset of PD. DJ-1 has three cysteines at amino acid numbers 46, 53, and 106 (C46, C53, and C106, respectively). Although oxidation of C106 is necessary for DJ-1 to exert its activity [12-15], further oxidation of C106 is thought to render DJ-1 inactive [16,17], and such oxidized DJ-1 has been observed in patients with the sporadic form of PD and Alzheimer disease [18,19].

We have shown that administration of DJ-1 protein dramatically reduced dopaminergic cell death and restored locomotion defect in PD model rats into which 6-hydroxydopamine (6-OHDA) had been injected [20] and that intrastriatal injection of DJ-1 markedly reduced infarct size in cerebral ischemia in rats [21], suggesting that DJ-1 is a pharmaceutical target for PD and cerebral ischemia. Another group also reported protective activity of DJ-1 against stroke [22]. Furthermore, we identified compounds that bind to the C106 region of DJ-1, and these compounds including compounds A and B, like DJ-1 protein, prevented oxidative stress-induced dopaminergic cell death and restored locomotion defect in PD model rats and also reduced infarct size in cerebral ischemia in rats [23-25]. These compounds were found by screening the University Compound library, which contains approximately 30,000 compounds.

In this study, we further screened DJ-1-binding compounds from the Zinc compound library that contains approximately 2,500,000 compounds. Of the compounds identified, compound-23 (comp-23) protected oxidative stress-induced cell death both in cultured cells and in PD and ischemia model rats and mice, and the protective activity of comp-23 seemed to be stronger than that of compound B.

## Results

### Isolation of a DJ-1-binding compound

**Figure 1:**
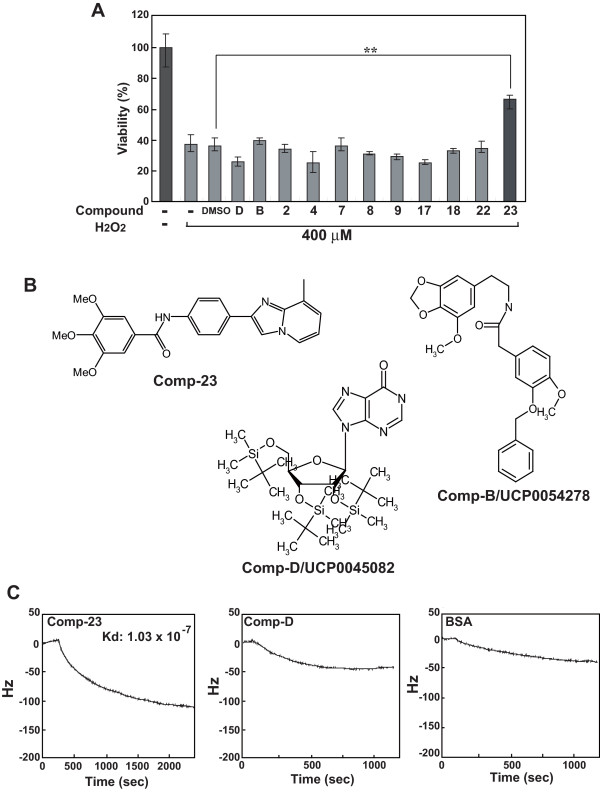
**Identification of DJ-1-binding compounds and effects of DJ-1-binding compounds on oxidative stress-induced cell death**. (A) SH-SY5Y cells were pretreated with 1 μM of each compound for 20 hours and then treated with H_2_O_2 _for 3 hours, and cell viability was measured by an MTT assay. ''-'' indicates cells not treated with compounds. Significance: ** *P *< 0.01 versus vehicle (DMSO) control without compounds. (B) Chemical structures of compound-23, -B and -D. (C) Binding of compounds to DJ-1 was examined by using a quartz crystal microbalance as described in Methods. Hz indicates decreased frequency of a sensor chip.

We have previously reported the isolation of DJ-1-binding compounds *in silico *using a Fujitsu Bioserver from a compound library, which is organized by the University Compound Project at the Foundation for Education of Science and Technology and contains approximately 30,000 compounds [23]. Based on the X-ray crystal structures of DJ-1 [26,27], compounds binding to the C106 region of DJ-1 were identified. In this study, we screened DJ-1-binding compounds *in silico *from the Zinc compound library that contains approximately 2,500,000 compounds using the same method as that described previously [23]. Twenty-five compounds whose docking score toward DJ-1 was less than -100 Kcal/mole were obtained. The effects of candidate compounds on oxidative stress-induced cell death were examined. Human dopaminergic neuroblastoma cell line SH-SY5Y cells were incubated with 1 μM of each compound for 20 hours and then treated with 400 μM H_2_O_2 _for 3 hours, and cell viability was measured by an MTT assay (Figure 1A). Results of some compounds were shown. Cell death induced by addition of H_2_O_2 _was significantly inhibited only by addition of compound-23 (comp-23) under this condition, and the other compounds, including compound B (comp-B) that was reported previously [23], had a little effect against cell death induced by less than 400 μM H_2_O_2_. Therefore, we concentrated on analyses of comp-23 in further study. Structures of comp-23 and comp-B are shown in Figure 1B.

Binding of comp-23 to DJ-1 was confirmed by using a quartz crystal microbalance in which compound-23, compound D (Figure 1B) or bovine serum albumin (BSA) was fixed on a sensor chip and recombinant DJ-1 was applied. Compound D is a negative control compound whose docking score toward DJ-1 was more than +200 Kcal/mole. As shown in Figure 1C, comp-23 bound to DJ-1, and compound D and BSA hardly bound to DJ-1. The binding constant (Kd) of comp-23 to DJ-1 is calculated to be 1.03 × 10^-7 ^M.

### Effects of DJ-1-binding compound-23 on oxidative stress-induced cell death and ROS production

**Figure 2:**
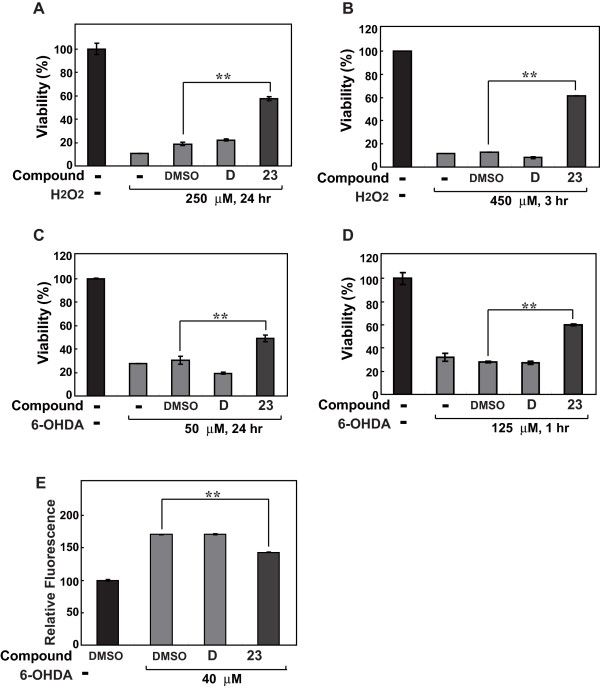
**Effects of DJ-1-binding compound-23 on oxidative stress-induced cell death**. (A-D) SH-SY5Y cells were pretreated with 1 μM of comp-23 for 20 hours and then treated with 250 μM H_2_O_2 _for 24 hours (A) and 450 μM H_2_O_2 _for 3 hours or with 50 μM 6-OHDA for 24 hours (C) and 125 μM 6-OHDA for 1 hour, and cell viability was measured by an MTT assay. "-'' indicates cells not treated with comp-23. Significance: ** *P *< 0.01 versus vehicle (DMSO) control without compound. (E) SH-SY5Y cells were pretreated with 1 μM of comp-23 for 20 hours, treated with 5 μM DCFH-DA for 10 min at 37°C, and then treated with 40 μM 6-OHDA for 10 min. The amounts of ROS in cells were measured using a fluorescence spectrophotometer.

The effect of comp-23 on oxidative stress-induced cell death was examined. SH-SY5Y cells were incubated with 1 μM comp-23 for 20 hours and then treated with 250 μM H_2_O_2 _for 24 hours or 450 μM H_2_O_2 _for 3 hours or with 50 μM 6-OHDA for 24 hours or 125 μM 6-OHDA for 1 hour, and cell viability was measured by an MTT assay (Figures 2A-D). Without the compound, 90-70% of the cells died and vehicle (DMSO) control of cells had little effect on protection against cell death. With comp-23, on the other hand, cell death induced by addition of H_2_O_2 _or 6-OHDA was significantly inhibited. Compound D had little effect. It should be noted that comp-23 at doses used in this study had no toxicity against culture cells.

The effect of comp-23 on production of reactive oxygen species (ROS) was then examined. SH-SY5Y cells were pretreated with 1 μM comp-23 for 20 hours and then treated with DCFA-DA and exposed to 40 μM 6-OHDA for 10 min. ROS were then measured by using a fluorescence spectrophotometer. As shown in Figure 2E, comp-23, but not comp-D, significantly reduced the level of ROS in cells that had been treated with 6-OHDA compared to that in vehicle-control cells.

**Figure 3:**
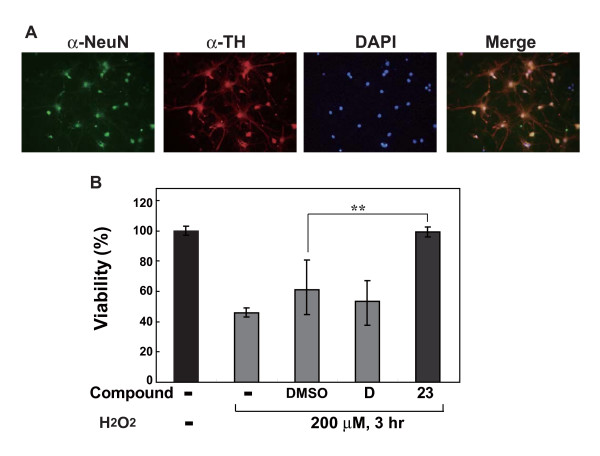
**Effects of compound-23 on oxidative stress-induced death of rat mesencephalic neurons**. (A) Rat mesencephalic cultured neurons were fixed and immunostained by anti-NeuN and anti-TH antibodies. Cells were then stained with DAPI. The cells were then reacted with a rhodamine-conjugated anti-rabbit IgG or fluorescein isothiocyanate-conjugated anti-mouse IgG and observed under an All-in-on microscope. (B) Rat mesencephalic cultured neurons were treated with 1 μM of comp-23 for 20 hours and with 200 μM H_2_O_2 _for 3 hours, and cell viability was measured by an MTT assay. Significance: **P < 0.01 versus vehicle (DMSO) control without compound.

Primary neuronal cells of the ventral mesencephalon were prepared from rat embryos on the 17-19th days of gestation. To examine the presence of dopaminergic neurons in cell culture, cells were immunostained using anti-NeuN and anti-TH antibodies to identify all of the neurons and dopaminergic neurons, respectively, and cell nuclei were stained with DAPI. Primary neuronal cells were pretreated with 1 μM comp-23 for 20 hours and then treated with 200 μM H_2_O_2 _for 3 hours. The results showed that comp-23, but not comp-D, significantly reduced cell death (Figure 3B).

### DJ-1-specific reaction of compound-23

**Figure 4:**
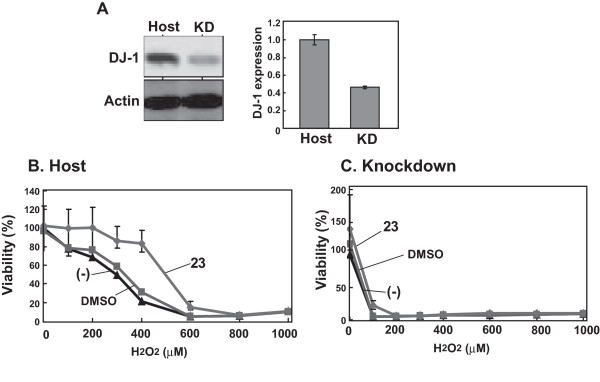
**DJ-1-specific action of DJ-1-binding compound-23**. (A) Cell extracts were prepared from SH-SY5Y and KD-SH-SY5Y cells, and proteins in the extracts were analyzed by Western blotting with anti-DJ-1 and anti-actin antibodies. After membranes had been reacted with respective secondary antibodies, bands were visualized and their intensities were quantified using an infrared imaging system (Odyssey, LI-COR). (B and C) SH-SY5Y (B) and KD-SH-SY5Y (C) cells were treated with 1 μM of comp-23 for 20 hours and with various concentrations of H_2_O_2 _for 3 hours, and cell viability was measured by an MTT assay.

To know the specificity of comp-23 to DJ-1, the effect of comp-23 on oxidative stress-induced cell death was examined using DJ-1-knockdown SHSY5Y cells (KD-SH-SY5Y cells) that had been established previously [15]. The expression levels of DJ-1 in KD-SH-SY5Y cells and parental SH-SY5Y cells (host) were examined by Western blotting with an anti-DJ-1 antibody and quantified by normalization of the level of DJ-1 compared to that of β-actin (Figure 4A). The results showed that about 60% of DJ-1 expression was knocked down in KD-SH-SY5Y cells. When SH-SY5Y and KD-SH-SY5Y cells were treated with 100 μM H_2_O_2 _for 3 hours, about 25% and 98% of the cells, respectively, died (Figures 3B and 3C), confirming that DJ-1-knockdown cells are more susceptible to oxidative stress than are parental cells as described previously [10,12,13,15]. Pretreatment of cells with com-23 for 20 hours before the addition of various concentrations of H_2_O_2 _significantly abrogated cell death of parental SH-SY5Y cells but not that of KD-SH-SY5Y cells (Figures 3B and 3C). These results clearly indicate that DJ-1-binding compound-23 functions in a DJ-1-dependent manner and that there is a threshold amount of DJ-1 for DJ-1-binding compounds to function in cells.

### Lack of scavenging activity for hydroxyl radical (^.^OH)

**Figure 5:**
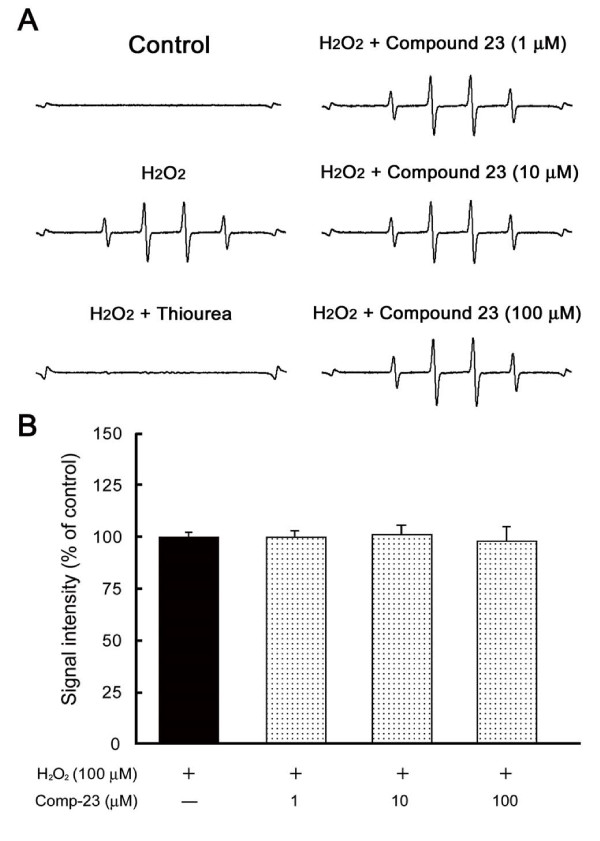
**ESR analysis**. (A) Typical ESR spectra of DMPO-OH spin adducts in the control (without H_2_O_2_), 100 μM H_2_O_2 _(with 25 μM Fe^2+^), H_2_O_2 _(with Fe^2+^) + 500 mM thiourea, and H_2_O_2 _(with Fe^2+^) + comp-23 at 1, 10 and 100 μM. (B) Semi-quantitative measurement of *in vitro *^.^OH generation. Each value is the mean ± SEM of eight determinations, based on H_2_O_2_/Fe^2+ ^as 100%.

Recent studies suggest that H_2_O_2 _is produced by mitochondrial dysfunction or autoxidation of dopamine and 6-OHDA, and then ^.^OH is easily generated in the presence of Fe^2+ ^[28,29]. It is known that ^.^OH is one of most potent neurotoxic factors in dopaminergic neurodegeneration. To clarify whether comp-23 can directly scavenge ^.^OH, we further performed electron spin resonance (ESR) analysis using a spin trapper, 5,5-dimethyl-1-pyrroline-*N*-oxide (DMPO). As an internal reference, Mn^2+ ^signal was detected as two small peaks at both edges (Figure 5A, Control). Although no marked signal was detected in the absence of Fe^2+^, four major peaks with an intensity ratio of 1:2:2:1 appeared at the mid-section between the Mn^2+ ^signal in the presence of H_2_O_2 _and Fe^2+ ^(Figure 5A, H_2_O_2_). This characteristic quartet signal was almost completely suppressed by thiourea (Figure 5A, H_2_O_2 _+ thiourea), a specific ^.^OH scavenger, suggesting that the quartet signal indicates DMPO-OH spin adduct. In contrast, H_2_O_2_-induced DMPO-OH signal could not be reduced by comp-23 even at a high concentration of 100 μM (Figure 5A). These results indicate that comp-23 is not a simple anti-oxidant.

### Effects of compound-23 on oxidation and dimer formation of DJ-1

**Figure 6:**
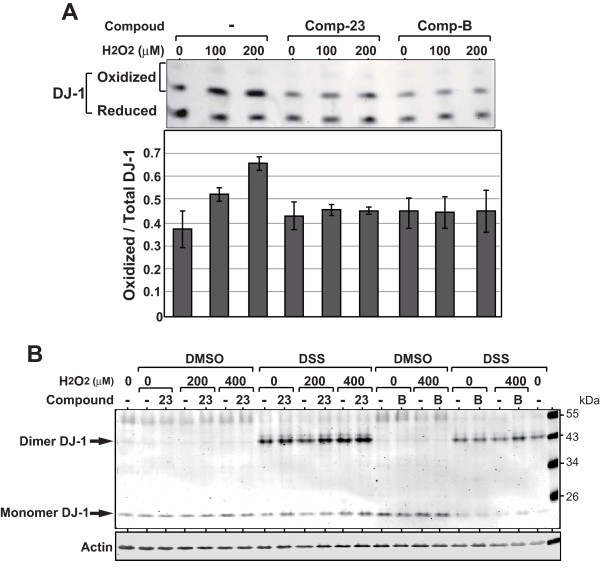
**Effects of compound-23 on excess oxidation and dimer formation of DJ-1**. (A) SH-SY5Y cells were incubated with 1 μM compound-23 or compound-B for 20 hours and then treated with various amounts of H_2_O_2 _for 15 min. Oxidation of DJ-1 in cells were analyzed by using isoelectric focusing phoresis followed by Western blotting with an anti-DJ-1 antibody (upper panel). Intensity of bands was quantified and ratio of oxidized DJ-1 to total DJ-1 is shown (lower panel). (B) SH-SY5Y cells were incubated with 1 μM compound-23 or compound-B for 20 hours and then treated with various amounts of H_2_O_2 _for 3 hours. Cells were then treated with 0.5 mM DSS or DMSO for 30 min, and proteins extracted from cells were analyzed by Western blotting with an anti-DJ-1 antibody.

We have reported that comp-B prevented excess oxidation of DJ-1 in cells that had been treated with H_2_O_2 _or 6-OHDA [23]. To examine whether this is true for comp-23, SH-SY5Y cells were first incubated with comp-23 or comp-B for 20 hours and treated with various amounts of H_2_O_2_. Oxidation of DJ-1 was analyzed by isoelectric focusing. As shown in Figure 6A, reduced and oxidized forms of DJ-1 were observed in cells in the absence of H_2_O_2_. After cells were treated with H_2_O_2_, the level of oxidized DJ-1 increased in cells that had not been treated with compound. No or little increase of the oxidized DJ-1 level was, on the other hand, observed in cells that had been incubated with comp-23 or with comp-B, indicating that comp-23, like comp-B, prevents excess oxidation of DJ-1.

Since DJ-1 works as dimer, the effect of comp-23 on dimer formation of DJ-1 was examined. SY-SY5Y cells were incubated with 1 μM comp-23 or with 1 μM comp-B for 20 hours, treated with various amounts of H_2_O_2 _for 3 hours and then treated with disuccinimidyl suberate (DSS) or with dimethyl sulfoxide (DMSO) as a vehicle control. Proteins extracted from cells were analyzed by Western blotting with an anti-DJ-1 antibody (Figure 6B). The results showed that the levels of dimmer DJ-1 observed in DSS-treated cells were not changed in the presence or absence of DJ-1-binding compounds, indicating that both comp-23 and comp-B do not affect dimer formation of DJ-1.

### Effects of compound-23 on oxidative stress-induced cell death and movement defect in Parkinson's disease model rats

**Figure 7:**
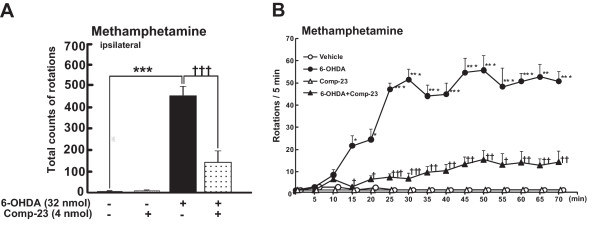
**Changes in methamphetamine-induced rotational behaviour in 6-OHDA-injected rats that had been co-injected or not co-injected with DJ-1-binding compound-23**. Rats were simultaneously injected with the vehicle (each n = 4) or 32 nmol of 6-OHDA (each n = 6) in the presence (n = 6) or absence (n = 4) of 4 nmol of DJ-1-binding compound-23 (comp-23) in a final volume of 4 μL sterilized physiological saline containing 0.02% ascorbic acid and 1% DMSO. Rotational behaviour was assessed at 7 days after 6-OHDA injection. The number of full body turns in the ipsilateral direction was counted for 70 min after the administration of methamphetamine (2.5 mg/kg, i.p.). Each value is the mean ± SEM. Significance (Bonferroni/Dunn *post hoc *comparisons after ANOVA in A; Student's *t*-test in B): *P < 0.05, **P < 0.01, ***P < 0.001 vs. vehicle control rats. †*P *< 0.05, ††*P *< 0.01, †††*P *< 0.001 versus 6-OHDA-injected rats.

**Figure 8:**
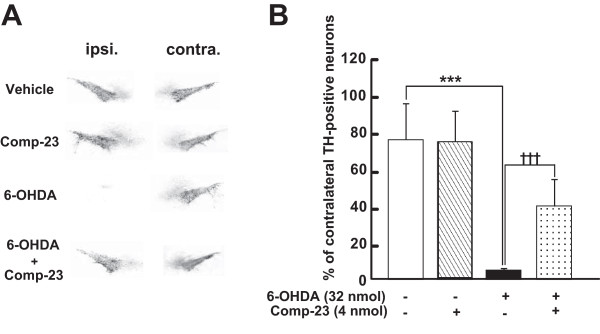
**Semi-quantitative analysis of dopaminergic neurons in the substantia nigra**. Co-administration of 6-OHDA and DJ-1-binding compound-23 (comp-23) was performed in rats injected with 6-OHDA into the left substantia nigra. After 10 days, treated rats were fixed and brain slices were prepared. (A) Midbrain slices were immunostained by anti-TH antibody. (B) 100% is the number of TH-immunopositive neurons in the contralateral substantia nigra (naive hemisphere). Each value is the mean ± SEM of TH-immunopositive neurons in ipsilateral nigral sections from treated rats (each group, n = 4-6). Significance (Bonferroni/Dunn *post hoc *comparisons after ANOVA): ***P < 0.001 versus vehicle control rats. †††*P *< 0.001 vs. 6-OHDA-injected rats.

**Figure 9:**
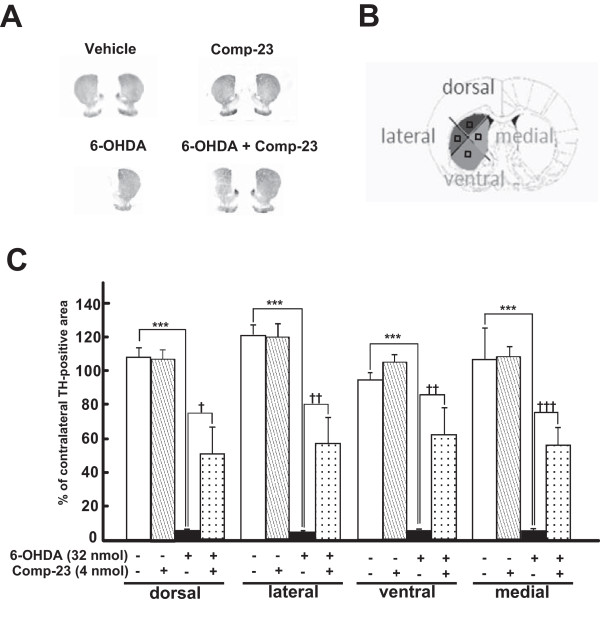
**Changes in TH immunoreactive areas in hemiparkinsonian striata**. (A) Co-administration of 6-OHDA and DJ-1-binding compound-23 (comp-23) was performed in rats injected with 6-OHDA into the left substantia nigra. After 10 days, treated rats were fixed and brain slices were prepared. Striatal slices were immunostained by anti-TH antibody. (B and C) For the analysis of striatal TH-immunoreactive intensity, the striatum at 0.60 mm anterior from the bregma was divided into four topographical areas, including the dorsal (D), lateral (L), ventral (V) and medial (M) regions (B), and the optical density of immunoreactivities for TH (C) was measured. Each value is the mean ± SEM based on the TH-immunoreactive intensity in ipsilateral striatal slices from treated rats (each group, n = 4-6). Significance (Bonferroni/Dunn test): ***P < 0.001 versus vehicle control rats. †††*P *< 0.05, †††*P *< 0.01, †††*P *< 0.001 vs. 6-OHDA-injected rats.

To examine the effect of DJ-1-binding comp-23 on PD phenotypes *in vivo*, we used PD model rats in which 6-OHDA was stereotaxically microinjected into the unilateral (left) mesencephalon. Administration of methamphetamine to animals induced movement ipsilateral to the injection site, and the rotational behavior was significantly reduced by coadministration of comp-23 at 7 days after injection (Figure 7). The total number of rotations of rats (Figure 7A) and number of rotations during the course of administration of methamphetamine (Figure 7B) were significantly reduced. As shown in Figure 8A, TH-immunopositive neurons were obviously preserved in the ipsilateral substantia nigra pars compacta (SNpc) of comp-23-treated animals compared to those in animals injected with 6-OHDA alone at 10 days post-lesion. Semi-quantitative analysis of nigral TH-immunopositive neurons showed that while microinjection of 6-OHDA alone caused a significant loss of dopaminergic neurons (5 ± 2% survival rate), loss of dopaminergic neurons was significantly inhibited by simultaneous administration of comp-23 (60 ± 13%) (Figure 8B). Comp-23 alone did not affect TH-immunoreactivity in the Snpc that had not been injected with 6-OHDA (Figure 8). In the ipsilateral striatum, although TH immunoreactivity almost completely disappeared in rats injected with 6-OHDA alone, TH immunoreactivity was restored by coadministration of comp-23 (Figure 9A). The intensity of TH-immunoreactivity in the striatal quadrants, including the dorsal, medial, lateral and ventral parts (Figure 9B), was significantly increased in comp-23-treated animals compared to the intensity in 6-OHDA-treated animals (Figure 9C).

### Effect of compound-23 on infarct size in focal cerebral ischemia and reperfusion in rats in a dose-dependent manner

**Figure 10:**
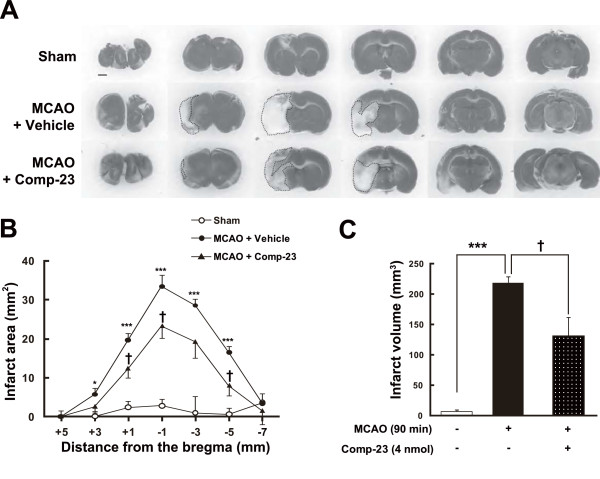
**Intrastriatal injection of DJ-1-binding compound-23 (comp-23) reduces infarct size after focal cerebral ischemia and reperfusion**. (A) Representative photographs showing coronal brain sections at +3, +1, -1, -3, -5, and -7 mm anterior-posterior from the bregma with TTC staining at 1 day after MCAO in sham-operated rats (n = 5) and MCAO-ischemic rats injected with sterilized physiological saline in the presence of the vehicle (4 μL, 1% DMSO, n = 5) or comp-23 (4 nmol/4 μL containing 1% DMSO, n = 4), at 30 min before MCAO (90 min). Scale bar: 1 mm (Sham in A). (B and C) Quantitative analysis of infarct area (B) and volume (C). Data are means ± SEM. Significance (Student's *t*-test in B; Bonferroni/Dunn *post hoc *comparisons after ANOVA in C): *P < 0.05, ***P < 0.001 versus sham-operated rats. †*P *< 0.05, †††*P *< 0.001 versus vehicle-injected rats.

**Figure 11:**
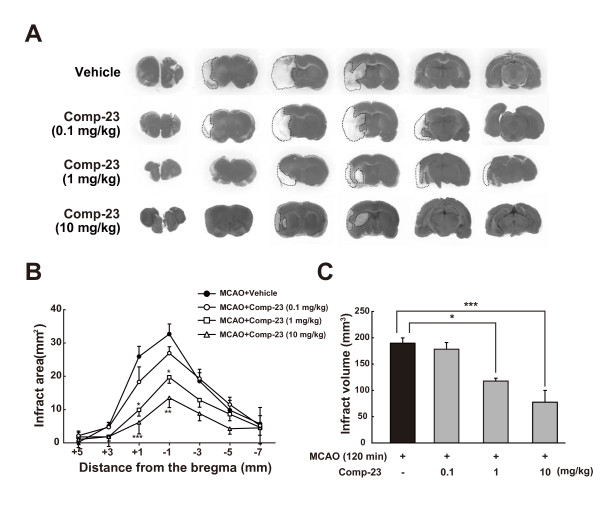
**Intraperitoneal administration of DJ-1-binding compound-23 (comp-23) reduces infarct size after focal cerebral ischemia and reperfusion**. (A) Representative photographs showing coronal brain sections at +3, +1, -1, -3, -5, and -7 mm anterior-posterior from the bregma with TTC staining at 1 day after MCAO in sham-operated rats (n = 6) and MCAO-ischemic rats intraperitoneally administered sterilized physiological saline in the presence of the vehicle (1% DMSO, n = 8) or comp-23 (0.1, 1 and 10 mg/kg containing 1% DMSO, n = 5 in each group), before 10 min and after 2 h of the reperfusion from 120-min MCAO. Scale bar: 1 mm (Sham in A). (B and C) Quantitative analysis of infarct area (B) and volume (C). Data are means ± SEM. Significance (Student's *t*-test in B; Bonferroni/Dunn *post hoc *comparisons after ANOVA in C): ***P < 0.001 versus sham-operated rats. †*P *< 0.05, †††*P *< 0.001 versus vehicle-injected rats.

Comp-23 was microinjected intrastriatally into the left striatum of rats, and left middle cerebral artery occlusion (MCAO) for 90 min and reperfusion were performed at 30 min after microinjection of comp-23. As shown in Figure 10A, although a marked regional loss of 2,3,5-triphenyltetrazolium chloride (TTC)-staining occurred in the ipsilateral cerebral cortex and striatum in vehicle-injected rats at 24 hours after MCAO, the area of TTC staining lost was smaller with microinjection of comp-23. In quantitative analysis, each infarct area was smaller and the total infarct volume was significantly reduced by the administration of comp-23 compared with that in vehicle-injected rats (Figures 10B and 10C). Thus, comp-23 exhibits neuroprotective effects by direct microinjection into the striatum of brain ischemic rats (Figure 10). Therefore, we further examined whether or not peripheral administration of comp-23 induces neuroprotection. Before and after 120-min MCAO, rats were intraperitoneally administered comp-23. Subsequently, we assessed the neuroprotective effect. As shown in Figure 11, focal ischemia-induced neurodegeneration was also prevented by peripheral administration of comp-23 in a dose-dependent manner. These results indicate that comp-23 has neuroprotective activity against oxidative stress-induced stroke and Parkinson's disease model rats.

### Effect of peripheral administration of compound-23 on rotenone-induced movement dysfunction in mice

**Figure 12:**
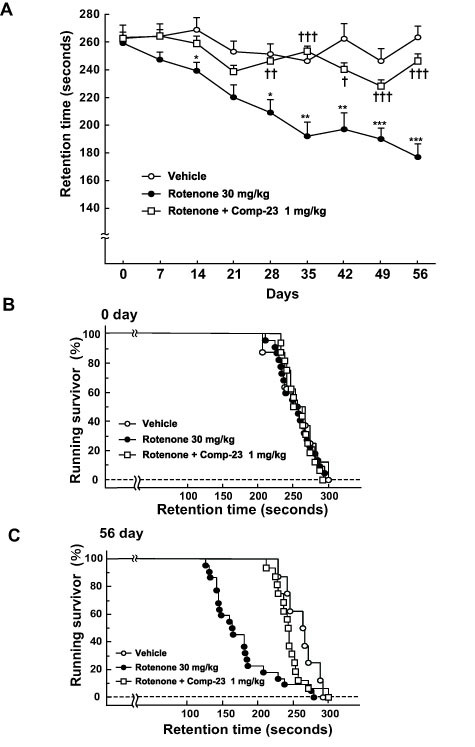
**Suppression of rotenone-induced behavioral dysfunction by DJ-1-binding compound-23**. Rotenone (suspended in 0.5% CMC) was orally administered to C57BL/6 mice at a dose of 30 mg/kg per day for 56 days. In addition, we injected mice with vehicle (closed circles; 1% DMSO, n = 22) or comp-23 (open squares; 1 mg/kg *i.p.*, n = 16) once a day for 56 days, 30 min before the oral administration of rotenone. The vehicle control mice (open circles; n = 8) were received 0.5% CMC (*p.o.*) and 1% DMSO (*i.p.*) for 56 days. (A) The rota-rod test was performed every week. The speed of the rotating rod was accelerated in a stepwise manner (2 r.p.m, steps at intervals of 30 sec). Mice that had received oral rotenone showed significant motor dysfunction. This rotenone-induced dysfunction was significantly restored by administration of comp-23. Significance: *P < 0.05, **P < 0.01, ***P < 0.001 vs. vehicle. †*p *< 0.05, ††*p *< 0.01, †††*p *< 0.001 vs rotenone alone. (B and C) Time-dependent changes in the percentage (%) of mice remaining on the rotating rod at 0 (B) and 56 days (C). Mice that had received oral rotenone showed significantly greater motor dysfunction than those that had received vehicle at 56 days (p = 0.0016 by the log-rank test, C). Rotenone-induced motor dysfunction was significantly restored by administration of comp-23 (*p *= 0.0019 by the log-rank test, C).

Although 6-OHDA-microinhected rat PD model is useful in pharmacological screening of drugs, the blood-brain barrier (BBB) is broken by the direct microinjection into the substantia nigra (ventral mesencephalon). We have previously shown that chronic oral administration to C57BL/6 mice with rotenone (a selective inhibitor for mitochondrial complex I) at 30 mg/kg for 28-56 days selectively induced nigrostriatal dopaminergic neurodegeneration and motor deficits, and increased the cytoplasmic accumulation of α-synuclein in surviving dopaminergic neurons, similar to the early stage of PD neuropathological episodes [30,31]. To investigate whether peripheral administration of comp-23 protects motor function from damage caused by the chronic oral administration of rotenone (30 mg/kg *p.o. *once a day for 56 days), we treated C57BL/6 mice with comp-23 (1 mg/kg *i.p. *once a day for 56 days) 30 min before the oral administration of rotenone. To identify deficits in motor coordination, rotenone-treated mice were tested weekly on the accelerating rota-rod. Under this condition, vehicle-treated control mice usually remained on the rota-rod for over 200 sec under stepwise acceleration. Rotenone-treated mice showed marked reduction in endurance time and in the percentage of mice remaining on the rota-rod (running survivors). In contrast, comp-23 (1 mg/kg *i.p.*) provided a significant functional recovery of the retention time on the rota-rod (Figure 12). Thus, chronic peripheral administration of comp-23 improves rotenone-induced Parkinsonian motor dysfunction.

## Discussion

In this study, we identified a new DJ-1-binding compound, compound-23 (comp-23), from the Zinc compound library, and we found that comp-23 prevented oxidative stress-induced cell death both in cultured cells and in PD and ischemia model rats and mice. Comp-23 prevented cell death even at a high concentration of H_2_O_2_, a condition in which DJ-1-binding compound B did not show protective activity against cell death, suggesting that activity of comp-23 is stronger than that of compound B at least in cultured cells. Structures of comp-23 and comp-B appear similar at a glance but are clearly different, especially in the position of an amino group and benzene ring. Since the X-ray co-crystal structure of DJ-1 with compound B has not yet been elucidated, an exact binding structure of compound B within DJ-1 is not known at present. Determination of the structure-activity relationships between DJ-1 and DJ-1-binding compounds will be necessary to establish DJ-1-binding compounds that are more effective than compounds B and 23. The Zinc compound library used in this study is freely available. If other libraries are used for screening of DJ-1-binding compounds, novel compounds might be obtained.

Although comp-23 lacks direct scavenging activity against ^.^OH (Figure 5), comp-23 protected SH-SY5Y cells and primary rat neurons from oxidative stress-induced cell death (Figures 1, 2, and 3). Since comp-23 did not show a protective effect against oxidative stress-induced cell death in DJ-1-knockdown SH-SY5Y cells (Figure 4), comp-23 works in a DJ-1-dependent manner. Since a residual amount of DJ-1 was still expressed in DJ-1-knockdown SH-SY5Y cells, no protective activity of comp-23 in DJ-1-knockdown cells suggests that there is a threshold amount of DJ-1 for DJ-1-binding compounds to function in cells. Comp-23 prevented dopaminergic cell death both in the substantia nigra and striatum in 6-OHDA-administered PD model rats, resulting in suppression of locomotion defect of rats (Figures 7, 8, 9, 10, 11). Since a precursor of dopamine, inhibitors of dopamine degradation and dopamine releasers are used for PD therapy at present and since these drugs are used for symptomatic therapy, cell death progresses during treatment. In the present study, the intraperitoneal injection of comp-23 at before and after MCAO induced neuroprotection in a dose-dependent manner (Figure 11), and peripheral administration of comp-23 for 56 days prevented rotenone-induced Parkinsonian motor deficit (Figure 12). Based on these observations, we consider that comp-23 binds to endogenous DJ-1 protein after passing through the BBB and that this DJ-1-comp-23 complex shows the neuroprotective effect against ROS-mediated dopaminergic neurodegeneration. Thus, there is a possibility that chronic peripheral administration of comp-23 delays the progression of motor dysfunction in PD and/or brain stroke.

Comp-23 is not a simple anti-oxidant (Figure 5) and prevented excess oxidation of DJ-1 in cells that had been treated with various amounts of H_2_O_2 _(Figure 6A). Since excess oxidation of DJ-1 renders DJ-1 inactive, it is thought that comp-23 activates DJ-1 or maintains active forms of DJ-1, thereby affecting downstream targets of DJ-1. DJ-1, for instance, activates Nrf2, a master transcription factor of redox-related genes, by sequestering Keap1, a negative factor of Nrf2 [32], and also activates the PI3 kinase/AKT pathway by inhibiting PTEN, a negative effecter of the PI3 kinase/AKT pathway, through direct binding with PTEN [22,33,34]. Screening strategy is to identify compounds that bind to weakly oxidized DJ-1 with an SO_2_H form of C106 using a model of such an oxidized DJ-1. Since reduced DJ-1 and oxidized DJ-1 are unable to be separately purified due to technical problem at present, we are not able to determine which form of DJ-1 is bound by comp-23. In vitro binding assays showed that comp-23 bound to recombinant DJ-1 that contains equal molar ratio of reduced and oxidized DJ-1 (Figure 1C), suggesting that comp-23 binds to both reduced DJ-1 and oxidized DJ-1. Furthermore, we examined dimer formation of DJ-1 in the presence and absent of comp-23. The results showed that comp-23 did not affect dimer formation of DJ-1 (Figure 6B). Since DJ-1 works as dimer, it is thought that dimer DJ-1 complexed with comp-23 shows protective activity against oxidative stress-induced neurodegeneration.

Reactive oxygen species are massively produced in the brain after cerebral ischemia and reperfusion. The antioxidant edaravone (3-methyl-1-phenyl-2-pyrazolin-5-one) has been used as a brain protectant for stroke therapy and is effective within 24 hours after onset of stroke. It has been reported that DJ-1 immunoreactivity in human brain astrocytes is dependent on infarct presence and infarct age [35], that DJ-1 is expressed in motor neurons after transient spinal cord ischemia in rabbits [36] and that loss of DJ-1 increases the sensitivity to excitotoxicity and ischemia [27]. We and other group have reported that injection of DJ-1 or infection of DJ-1-containing virus reduced infarct size in cerebral ischemia in rats [21,22]. Furthermore, we have shown that administration of DJ-1-binding compound B also reduced infarct size of cerebral ischemia in rats [24]. It is therefore thought that, like a PD model, comp-23 maintains activated forms of DJ-1 to activate Nrf2 and the AKT pathway, leading to reduction of ROS and to promotion of cell growth in ischemia model rats.

## Conclusions

In this study, we identified a new DJ-1-binding compound, comp-23. Comp-23 prevented dopaminergic cell death in the substantia nigra and restored movement abnormality in 6-hydroxyldopamine-injected PD model rats and in rotenone-treated PD model mice. Comp-23 also reduced infarct size of cerebral ischemia in rats that had been induced by middle cerebral artery occlusion. Protective activity of comp-23 seemed to be stronger than that that of previously identified compound B at least in cultured cells. Com-23 will become a lead compound for PD and stroke.

## Methods

### Materials

N-[4-(8-methyl(4-hydroimidazo[1,2-a]pyridin-2-yl))phenyl](3,4,5-trimethoxyphenyl)carboxamide, which is DJ-1-binding compound-23 (comp-23), was synthesized and obtained by Enamine Ltd. (Kiev, Ukraine). 6-Hydroxydopamine (6-OHDA) and DCFH-DA were purchased from Sigma (St. Louis, MO, USA) and from Invitrogen (Carlsbad, CA, USA), respectively. Mouse anti-tyrosine hydroxylase (TH), chicken anti-TH and anti-NeuN antibodies were purchased from Sigma, Chemicon (Temecula, CA, USA) and Chemicon, respectively. The ABC Elite kit from Vector Laboratories (Burlingame, CA, USA) was used. Methamphetamine was obtained from Dainippon Sumitomo Pharmaceutical Co., Ltd. (Osaka, Japan).

### Cell culture

Human SH-SY5Y and its DJ-1-knockdown cells were cultured in Dulbecco's modified Eagle's medium (DMEM) with 10% calf serum. Establishment of DJ-1-knockdown SH-SY5Y cells was described previously [17].

### Screening of DJ-1-binding compounds

Information on the X-ray crystal structures of reduced DJ-1 and oxidized DJ-1 at C106 as an SO_2_H form was obtained from a web site (http://www.rcsb.org/pdb/). To obtain the structure of DJ-1 containing H_2_O, the X-ray crystal structure of DJ-1 was modified using BioMedCAChe software (Fijitsu, Tokyo, Japan). Compounds were screened by targeting C106 of this structure on FastDock software (Fijitsu) in BioServer hardware (Fujitsu) according to the manufacturer's protocol. Briefly, the BioServer hardware used is PC clusters with 40 core of CPU of Xeon5355 (Fujitsu), OS of Red Hat 3.4.5-2 (Linux version 2.6.9-34) and 1.0 TB Hard Disk. The other conditions were exactly the same as those described previously [23].

### Cell viability assay

Cells were cultured in a 96-well plate and treated with various amounts of hydrogen peroxide or 6-OHDA. Cell viability was then measured by a 3-(4,5-dimethylthiazol-2-yl)-2,5-diphenyltetrazolium bromide (MTT) assay using a cell counting kit -8 (DOJINDO, Osaka, Japan).

### Binding of compound-23 to DJ-1 by a quartz crystal microbalance

Fixation of compounds on a sensor chip of QCM (Affinix Q, Initium, Tokyo, Japan) was carried out as follows. The sensor chip was washed with a solution containing H_2_O_2 _and sulfonic acid (molar ratio = 1:3), and then it was incubated with 4 μL of 1 μM compound dissolved in chloroform until the solution had evaporated. To the sensor chips fixed with compounds in Affinix Q, 8 μL of 1 μg/μL DJ-1 was applied, and their frequency was measured according to the manufacturer's protocol.

### Primary neuronal culture of the ventral mesencephalon

Cultures of the rat mesencephalon were established according to methods described previously [37]. The ventral two-thirds region of the mesencephalon was dissected from rat embryos on the 17-19th days of gestation. The dissected regions included dopaminergic neurons from the substantia nigra and the ventral tegmental area but not noradrenergic neurons from the locus ceruleus. Neurons were dissociated mechanically and plated out onto 0.1% polyethyleneimine-coated 24-well plates at a density of 2.5 × 10^6 ^cells/well. The culture medium consisted of DMEM containing 10% fetal calf serum for 2 days and DMEM containing 2% B-27 supplement (Invitrogen) and 2 μg/mL aphidicolin (Sigma) without fetal calf serum from the third day onwards. The animals were treated in accordance with guidelines published in the NIH Guide for the Care and Use of Laboratory Animals. After fixation, cultured cells were incubated with chicken anti-TH (diluted at 1:200) and anti-NeuN (1:200) antibodies for 24 hours at 25°C. The cells were also stained with 4',6-diamidino-2-phenylindole (DAPI). The cells were then reacted with a rhodamine-conjugated anti-rabbit IgG or fluorescein isothiocyanate-conjugated anti-mouse IgG and observed under an All-in-on microscope (Biorevo BZ-9000, Keyence).

To examine the effects of DJ-1-binding compounds on oxidative stress-induced cell death, the cells were cultured in the presence or absence of 1 μM of each compounds for 20 hours and then treated with 200 μM H_2_O_2 _for 3 hours. Cell viabilities were then examined by an MTT assay.

### Detection of production of ROS

8 × 10^5 ^SH-SY5Y cells in a 96-well plate were pretreated with 1 μM of comp-23 for 20 hours and then treated with 40 μM 6-OHDA for 10 min after the addition of 10 μM DCFA-DA (Invitrogen) for 15 min. The amounts of ROS in cells were measured using a fluorescence spectrophotometer at extension of 485 nm and emission of 530 nm.

### Isoelectric focusing

SH-SY5Y cells were incubated with 1 μM compound-23 or compound-B for 24 hours and then treated with various amounts of H_2_O_2 _for 10 min. Proteins extracted from the cells were separated in the pH 5-8 range of isoelectric focusing phoresis gel, transferred to nitrocellulose membranes, and blotted with an anti-DJ-1 polyclonal antibody as described previously [10].

### Dimer formation

SH-SY5Y cells in 6-well plates were incubated with 1 μM compound-23 or compound-B for 20 hours and then treated with various amounts of H_2_O_2 _for 3 hours. Cells were then treated with 0.5 mM DSS or DMSO for 30 min, and proteins extracted from cells were analyzed by Western blotting with an anti-DJ-1 antibody.

### ESR spectrometry

The hydroxyl radical (^.^OH) was monitored by ESR spectrometry with 5,5-dimethyl-1-pyrroline-*N*-oxide (DMPO; Labotec Ltd., Tokyo, Japan), a spin trapper. In a final volume of 200 μL of 100 mM phosphate buffer (PB), comp-23 (1-100 μM) or thiourea (500 mM) was added to the reaction mixture containing diethylene-triamine pentaacetic acid (25 μM), FeSO_4 _(25 mM), H_2_O_2 _(100 μM), and DMPO (112.5 mM). These drugs and reagents were solubilized in Milli Q water. The reaction mixture was transferred to a flat quartz cuvette and placed in the cavity of an X-band JEOL RFR-30bRadical Analyzer system (JEOL Ltd., Tokyo, Japan). The ^.^OH, which was generated by Fenton's reaction between Fe^2+ ^and H_2_O_2_, was trapped by DMPO, and a stable adduct DMPO-OH was measured exactly 1 min after the addition of DMPO. The Mn^2+^-derived split signal was used as the internal standard. Typical instrumental stettings were as follows: incident-microwave of 4 mV, modulation-amplitude of 0.1 mT, time-constant of 0.10 s, and sweep rate of 5 mT/min.

### Hemiparkinsonian rats

Male Wistar rats (SLC, Shizuoka, Japan) weighing approximately 250 g were used. Rats were acclimated to and maintained at 23°C under a 12-hour light and dark cycle (light on 08:00-20:00 hours). All animal experiments were carried out in accordance with the National Institutes of Health Guide for the Care and Use of Laboratory Animals, and the protocols were approved by the Committee for Animal Research at Kyoto Pharmaceutical University. For stereotaxic microinjection, rats were anesthetized (sodium pentobarbital, 50 mg/kg, i.p.) and immobilized in a Kopf stereotaxic frame. Subsequently, rats were simultaneously injected with 6-OHDA (32 nmol/4 μL) in the presence or absence of comp-23 (4 nmol/4 μL), in a final volume of 4 μL of physiological saline containing 0.02% ascorbic acid (as a 6-OHDA stabilizer) and 1% dimethyl sulfoxide (DMSO, as a solvent for comp-23). As a vehicle control, sterilized physiological saline containing 0.02% ascorbic acid and 1% DMSO was injected without 6-OHDA. The intranigral injection coordinates 4.8 mm anterior-posterior, 1.8 mm left lateral, and 7.8 mm ventral from the bregma were taken from a rat brain atlas. Injection was performed by a motor-driven 10-μl Hamilton syringe using a 26-gauge needle. The infusion rate was 1 μL/min, and the injection needle was kept in place for a further 5 min after injection. At the end of the experiments, all rats were sacrificed for immunohistochemical assessments.

### Assay of rotational behavior

We used methamphetamine as a dopamine releaser [38]. Drug-induced rotational asymmetry was assessed in rotometer bowls as described previously [20,23,39]. Briefly, the number of full body turn rotations in the ipsilateral direction was counted after the administration of methamphetamine (2.5 mg/kg, i.p., for 70 min).

### Tissue preparation and immunohistochemistry

After assay of rotational behaviour, treated rats were perfused through the aorta with 150 mL of 10 mM PBS, followed by 300 mL of a cold fixative consisting of 4% paraformaldehyde in 100 mM phosphate buffer (PB) under deep anesthesia with pentobarbital (100 mg/kg, i.p.). After perfusion, the brain was quickly removed and postfixed for 2 days with paraformaldehyde in 100 mM PB and then transferred to 15% sucrose solution in 100 mM PB containing 0.1% sodium azide at 4°C. The brain was cut into 20-μm-thick slices using a cryostat and collected in 100 mM PBS containing 0.3% Triton X-100 (PBS-T). Brain slices were incubated with a mouse anti-TH antibody (1:10,000, dilution) for 3 days at 4°C. After several washes, sections were incubated with biotinylated anti-mouse IgG antibody (1:2,000), as appropriate, for 2 hours at room temperature. The sections were then incubated with avidin peroxidase (1:4,000; ABC Elite Kit; Vector Laboratories, Burlingame, CA, USA) for 1 hour at room temperature. All of the sections were washed several times with PBS-T between each incubation, and labeling was then revealed by 3,3'-diaminobenzidine (DAB) with nickel ammonium, which yielded a dark blue colour [20,23].

### Measurement of immunoreactive neurons and areas

The number of TH-immunopositive neurons in the substantia nigra and the optical density of TH-immunoreactive areas in the striatum were measured by a computerized image analysis system (WinRoof; Mitani, Tokyo, Japan) with a CCD camera (ProgRes 3008, Carl Zeiss, Jena, Germany) as described previously [20,23]. The number of TH-immunopositive neurons in the substantia nigra was counted bilaterally on six adjacent sections between 4.6 and 4.9 mm posterior from the bregma. For each animal, neuronal survival in the substantia nigra was then expressed as the percentage of TH-immunopositive neurons on the lesioned side, with respect to the contralateral, intact side; this approach was chosen to avoid methodological biases because of interindividual differences and is widely used to assess the extent of a 6-OHDA-induced lesion in the substantia nigra [40-42].

For the analysis of striatal TH-immunoreactive intensity, the striatum was divided into anatomo-functional quadrants encompassing the dorsal (D), lateral (L), ventral (V), and medial (M) regions [41,43] and the optical density was measured within a fixed box (0.5 × 0.5 mm) positioned approximately in the middle of these quadrantal parts. Immunoreactive intensity was expressed as percentage of the intensity recorded from the same area on the contralateral side [40,43,44]. Subsequently, the average of relative intensities in each quadrant was estimated from striatal slices (at 0.60 mm anterior from the bregma) and then statistical values were evaluated from treated rats.

### In vivo model of rat focal cerebral ischemia

Male Wistar rats weighing 260-300 g were used. Focal cerebral ischemia was induced by the intraluminal introduction of a nylon thread as described previously [21]. Briefly, animals were anesthetized with 4% halothane (Takeda Pharmaceutical, Osaka, Japan) and maintained on 1.5% halothane using a facemask. After a midline neck incision had been made, 20 mm of 4-0 nylon thread with its tip rounded by heating and coated with silicone (Xantopren M; Heraeus Kulzer, Hanau, Germany) was inserted into the left internal carotid artery (ICA) as far as the proximal end using a globular stopper. The origin of the middle cerebral artery (MCA) was then occluded by a silicone-coated embolus. Anesthesia was discontinued, and the development of right hemiparesis with upper limb dominance was used as the criterion for ischemic insult. After 90 or 120 min of MCA occlusion (MCAO), the embolus was withdrawn to allow reperfusion of the ischemic region via the anterior and posterior communicating arteries. Body temperature was maintained at 37-37.5°C with a heating pad and lamp during surgery. In the sham operation, a midline neck incision was made to expose the arteries, but the nylon thread was not inserted into the carotid artery.

### Intrastriatal drug administration to ischemic rats

Ninety-min-MCAO-ischemic rats (SLC, Shizuoka) were used. Under deep anesthesia (sodium pentobarbital, 50 mg/kg, i.p.), rats received a microinjection of comp-23 (4 nmol/4 μL) in the left striatum (coordinates: 1 mm anterior, 4 mm left lateral, and 5 mm ventral from the bregma). Sterilized physiological saline containing 1% DMSO was used as the vehicle control in a final volume of 4 μL. After 30 min, left MCAO for 90 min and reperfusion were performed.

### Intraperitoneal drug administration to ischemic rats

One hundred twenty-min-MCAO-ischemic rats were used. Animals were intraperitoneally administered with comp-23 (0.1, 1 and 10 mg/kg), before 10 min and after 2 hours of the reperfusion from MCAO. Sterilized physiological saline containing 1% DMSO was used as a vehicle control.

### Measurement of infarct volume in rat ischemic brain

At 24 hours after MCAO, brains were removed and cut into 2-mm-thick coronal sections. These sections were immersed in 2% solution of 2,3,5-triphenyltetrazolium chloride (TTC; Wako Pure Chemical Industries, Osaka, Japan) in saline at 37°C for 20 min and then fixed in 4% paraformaldehyde in 100 mM phosphate buffer (PB) at 4°C, and infarct areas and volumes were quantified.

### Rotenone-treated PD model mice and rota-rod test

Rotenone (Sigma, St. Louis, MO, USA) was administered orally once daily at a dose of 30 mg/kg for 56 days, as described previously [30,31]. Rotenone was suspended in 0.5% carboxymethyl cellulose sodium salt (CMC, Nacalai Tesque, Kyoto, Japan) and administered orally once daily at a volume of 5 mL/kg body weight. 0.5% CMC was administered orally as vehicle to control mice.

Behaviour of each mouse was assessed by the rota-rod test, as also described previously [30,31]. The rota-rod treadmill (accelerating model 7750, Ugo Basile, Varese, Italy) consists of a plastic rod, 6 cm in diameter and 36 cm long, with a non-slippery surface 20 cm above the base (trip plate). This rod is divided into four equal sections by five discs (25 cm in diameter), which enables four mice to walk on the rod at the same time. In the present study, the accelerating rotor mode was used (10-grade speeds from 2 to 20 r.p.m. for 5 min). The performance time was recorded while mice were running on the rod.

### Statistical evaluation

All data are presented as means ± standard error of the mean (SEM). The significance of differences was determined by one-way analysis of variance (ANOVA). Further statistical analysis for *post hoc *comparisons was performed using the Bonferroni/Dunn tests (StatView; Abacus Concepts, Berkeley, CA, USA). On the other hand, the significance of difference in rotation numbers/5 min and that of difference in areas of survival neurons in 6-OHDA-injected rats and MCAO-ischemic rats were determined by Student's *t*-test for single comparisons. Endurance performance (percentage of mice remaining on the rota-rod) was calculated by the Kaplan-Meier method. The statistical significance of differences was analyzed by the log-rank (Mantel-Cox) test.

## Abbreviations

The abbreviations used are PD: Parkinson's disease; comp-23: compound 23; 6-OHDA: 6-hydroxydopamine; MCA: middle cerebral artery; MCAO: MCA occlusion; ROS: reactive oxygen species; TH: tyrosine hydroxylase; MTT: 3-(4,5-dimethylthiazol-2-yl)-2,5-diphenyltetrazolium bromide.

## Competing interests

The authors declare that they have no competing interests.

## Authors' contributions

HA and SMMI-A conceptualized the study; YK, SW, MT, KT, TK, KT-N and HY carried out experiments; HM conducted the analyses and YK and HA wrote the manuscript. All authors read and approved the final manuscript.
